# Tax4Fun2: prediction of habitat-specific functional profiles and functional redundancy based on 16S rRNA gene sequences

**DOI:** 10.1186/s40793-020-00358-7

**Published:** 2020-05-18

**Authors:** Franziska Wemheuer, Jessica A. Taylor, Rolf Daniel, Emma Johnston, Peter Meinicke, Torsten Thomas, Bernd Wemheuer

**Affiliations:** 1 Evolution and Ecology Research Centre, School of Biological, Earth and Environmental Sciences, University of New South Wales, Sydney, NSW 2052 Australia; 2 Sydney Institute of Marine Science, Mosman, NSW 2088 Australia; 3 Centre for Marine Science and Innovation, School of Biological, Earth and Environmental Sciences, University of New South Wales, Sydney, NSW 2052 Australia; 4 Genomic and Applied Microbiology and Göttingen Genomics Laboratory, Institute of Microbiology and Genetics, University of Göttingen, Göttingen, Germany; 5 Department of Bioinformatics, Institute of Microbiology and Genetics, University of Göttingen, Göttingen, Germany

**Keywords:** Metagenomics, Functional predictions, 16S rRNA gene, Bioinformatics, Microbiome, Multifunctional redundancy, Ecosystem functioning

## Abstract

**Background:**

Sequencing of 16S rRNA genes has become a powerful technique to study microbial communities and their responses towards changing environmental conditions in various ecosystems. Several tools have been developed for the prediction of functional profiles from 16S rRNA gene sequencing data, because numerous questions in ecosystem ecology require knowledge of community functions in addition to taxonomic composition. However, the accuracy of these tools relies on functional information derived from genomes available in public databases, which are often not representative of the microorganisms present in the studied ecosystem. In addition, there is also a lack of tools to predict functional gene redundancy in microbial communities.

**Results:**

To address these challenges, we developed Tax4Fun2, an R package for the prediction of functional profiles and functional gene redundancies of prokaryotic communities from 16S rRNA gene sequences. We demonstrate that functional profiles predicted by Tax4Fun2 are highly correlated to functional profiles derived from metagenomes of the same samples. We further show that Tax4Fun2 has higher accuracies than PICRUSt and Tax4Fun. By incorporating user-defined, habitat-specific genomic information, the accuracy and robustness of predicted functional profiles is substantially enhanced. In addition, functional gene redundancies predicted with Tax4Fun2 are highly correlated to functional gene redundancies determined for simulated microbial communities.

**Conclusions:**

Tax4Fun2 provides researchers with a unique tool to predict and investigate functional profiles of prokaryotic communities based on 16S rRNA gene sequencing data. It is easy-to-use, platform-independent and highly memory-efficient, thus enabling researchers without extensive bioinformatics knowledge or access to high-performance clusters to predict functional profiles. Another unique feature of Tax4Fun2 is that it allows researchers to calculate the redundancy of specific functions, which is a potentially important measure of how resilient a community will be to environmental perturbation. Tax4Fun2 is implemented in R and freely available at https://github.com/bwemheu/Tax4Fun2.

## Background

Microorganisms are key players in ecosystem functioning [1, 2]. For example, host-associated microorganisms significantly contribute to the health of their host organism, e.g., by providing essential nutrients or by enhancing the host’s resilience to pathogens or pests [3–5]. High-throughput sequencing of 16S rRNA genes is a powerful and widely used tool to study the taxonomic or phylogenetic composition of microbial communities in a variety of marine [6–8], terrestrial [9, 10] and host-associated [11–13] environments. However, numerous questions in biogeochemistry and ecology require knowledge of microbial community function, rather than, or in addition to, the taxonomic or phylogenetic composition [14]. Investigating the composition of microbial communities and their functional capabilities is of particular importance when the effect of changing environmental conditions or anthropogenic perturbations on ecosystem services is being assessed [15].

Many ecosystems are threatened by environmental perturbations. A key question in microbial ecology is whether, and to what degree, microbial communities contain functionally redundant members that may provide stability to ecosystem processes in the face of these perturbations [8, 16–18]. It has been proposed that the evaluation of multiple functions provide a more comprehensive picture on the role of biodiversity for maintaining ecosystem functions [19]. However, the simultaneousness assessment of multiple functions is time-consuming [20]. Some researchers have started to address this question by developing metatranscriptome-based [20] or metagenome-based [21] approaches for quantifying the multifunctional redundancy of microbial communities [20, 21]. To the best of our knowledge, no tools are currently available to provide a standardized method for the simultaneous calculation of functional redundancy for multiple functions.

In past years, several freely available tools including PICRUSt [22], Tax4Fun [23], Piphillin [24], Faprotax [25] and paprica [26] have been developed for the prediction of functional profiles inferred from 16S rRNA gene sequence data. Although these tools cannot replace the functional assessment via metagenomic shotgun sequencing, they have provided unique insights into functional capabilities of prokaryotic communities in diverse habitats, such as microbial mats [27], soil [28–31], marine seawater [25, 26, 32, 33], sediment [8, 34], rumen [35, 36] and the plant endosphere [37, 38].

The predictive power of these tools relies on functional information derived from genomes available in public databases. This information is used to predict functional profiles even if no close reference genomes are present in these databases. However, available genomes do not necessarily represent the functional diversity present in the ecosystem investigated. This problem has motivated the development of predictive tools specific for the rumen microbiome [35] or marine microorganisms [25]. Given the rapidly increasing number of available genomes, in particular through metagenome-assisted genome binning [39], and that many research groups have access to unpublished, habitat-specific genomic information, the incorporation of such data is likely to enhance the accuracy of functional inferences.

Here, we introduce Tax4Fun2, a new and improved version of Tax4Fun [23]. Tax4Fun2 is platform-independent, user-friendly and highly memory-efficient. It can incorporate habitat-specific and user-defined data. Although Tax4Fun2 focuses on prokaryotic data, eukaryotic data can also be incorporated. We show that the incorporation of habitat-specific data improves the practical utility of Tax4Fun2 for microbiome datasets from a wide range of ecosystems. Comparative analysis further shows that Tax4Fun2 has a higher accuracy than PICRUSt [22] and Tax4Fun [23]. Another unique feature of Tax4Fun2 is that it enables researchers to calculate the functional redundancy of multiple functions, which is critical for the prediction of how likely a specific ecosystem function is to be lost during environmental perturbation. This information might be important in ecosystem biomonitoring and the prioritisation of environmental management actions.

### Implementation

Tax4Fun2 is provided as an R [40] package with a current default reference dataset build from 12,377 archaeal and bacterial genomes available through the NCBI RefSeq database. The current version is 1.1.5. Tax4Fun2 is platform-independent and enables researchers without extensive bioinformatics knowledge to predict functional profiles in an efficient and user-friendly manner. In the following sections, we provide further details in the generation of the reference data and the technical implementation of Tax4Fun2.

#### Tax4Fun2 reference data

Tax4Fun2 is supplied with two reference datasets (Ref99NR and Ref100NR) referring to the similarity threshold used during clustering of the 16S rRNA gene sequences. Each dataset consists of an association matrix with 16S rRNA gene reference sequences associated with functional reference profiles (number of entries in the full association matrix: 4584 and 18,479 for Ref99NR and Ref100NR, respectively). Due to its smaller size, the Ref99NR database is less hardware-demanding and predictions are generated faster compared to the Ref100NR. In order to obtain the default reference data, we included the function *buildReferenceData,* which will automatically download and build the reference data.

The reference datasets were generated as follows: we downloaded all complete genomes and all genomes with the status ‘chromosome’ from NCBI RefSeq (assessed on 18 August 2018), resulting in 275 archaeal and 12,102 bacterial genomes. Barrnap version 0.9 (https://github.com/tseemann/barrnap) was used to identify and extract all 16S rRNA gene sequences. All rRNA gene sequences were subsequently concatenated into a single file, sorted by decreasing length and clustered using the UCLUST algorithm implemented in USEARCH version 10.240 [41] at 99 and 100% sequence similarity, respectively. The longest sequence of each cluster serves as the 16S rRNA reference sequence. Functional profiles for each genome were generated as follows: open-reading-frames were predicted with prodigal version 2.6.3 [42]. Functional profiles were calculated based on obtained protein sequences with UProC version 1.2.0 [43] using the KEGG Orthology (KO) database for prokaryotes (July 2018 release; [44]) as reference. To account for differences in rRNA copy numbers, functional profiles were normalized by the number of 16S rRNA genes identified in each genome. Due to the heterogeneity of 16S rRNA genes within a genome, the functional reference profile for each 16S rRNA reference sequence was generated based on the 16S rRNA clustering results; a single functional reference profile is the average normalized functional profile of each genome with at least one 16S rRNA gene affiliated to the cluster. If more than one 16S rRNA gene sequence of a genome was assigned to a cluster, the normalized profile of the genome was multiplied by the number of 16S rRNA genes affiliated to the cluster before calculating the mean profile. To calculate phylogenetic distances, two phylogenetic trees (one tree for each reference dataset) are included in the reference data. These were generated as follows: all 16S rRNA reference sequences were aligned with SINA version 1.2.11 [45] against the latest Silva ARB release (SILVA_132_SSUREF_NR99) [46]. The phylogenetic tree was subsequently calculated using RaxML version 8.2.11 [47] under a GTRGAMMA model and a random seed of 12,345.

#### Extending the reference data of Tax4Fun2 using specific genomes

**Fig. 1:**
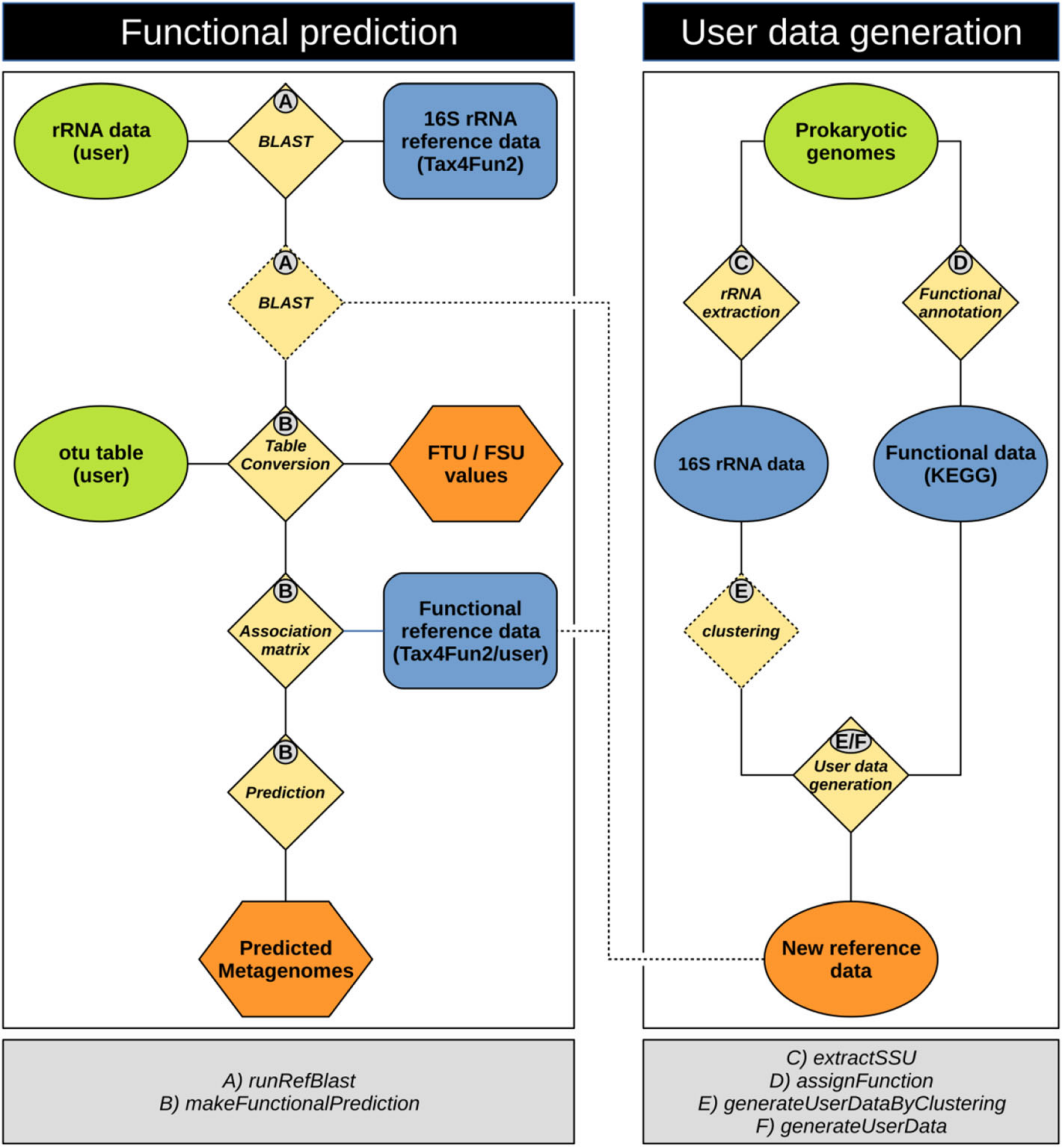
The Tax4Fun workflow. 16S rRNA gene sequences are initially aligned against the reference sequences provided with Tax4Fun2 to identify the nearest neighbour. If user-defined data is supplied, the 16S rRNA gene sequences are additionally aligned against the sequences added by the user. Nearest neighbours in the user data will be preferred if both search attempts result in significant hits. The OTU abundances for each sample are summarized based on the results from the nearest neighbour search. An association matrix (AM) containing the functional profiles of those references identified in the 16S rRNA search is generated. The summarized abundances and the functional profiles stored in the AM are merged and a metagenome is predicted for each sample. FTU and FSU values are provided as a log file. To include user data and generate a habitat-specific data set, Tax4Fun2 provides functions to functionally annotate prokaryotic genomes and to extract 16S rRNA gene sequences. User-defined reference data sets are subsequently generated and can be included in the prediction. If large amounts of genomes are provided, extracted 16S rRNA gene sequences can be optionally clustered using the uclust algorithm

The predictive power of available tools, such as PICRUSt or Tax4Fun, is limited by the number of genomes available in public databases that also need frequent updates. Moreover, available genomes do not necessarily represent the functional diversity present in the ecosystem investigated. In Tax4Fun2, we address these issues by allowing users to build their own habitat-specific reference data sets (Fig. 1). In order to build such a dataset, the user needs to provide a set of genomes. To assist with the extraction of 16S rRNA sequences and the functional annotation of these genomes, we implemented two functions in the Tax4Fun2 package: *extractSSU* and *assignFunctions*. Ribosomal RNA sequences (16S rRNA or 18S rRNA) are identified by BLAST search using the SILVA SSURef database version 132 [46] (preclustered at 90% with uclust). Functional profiles are generated by BLASTp with diamond against the KEGG KO database [48]. Protein sequences are predicted prior to functional annotation using prodigal version 2.6.3 [42]. Currently, the functional annotation is only available for prokaryotes but will be extended to eukaryotes in later versions. The extracted rRNA sequences and functional profiles are subsequently used to build reference data sets using the *addUserDataByClustering* or *addUserData* functions (Fig. 1). vsearch is required to use the first function [49]. vsearch is freely available at https://github.com/torognes/vsearch and included in Tax4Fun2 as part of the reference data. The latter function bypasses the clustering step with vsearch and is recommended if only a small number of distinct genomes shall be used as reference data.

#### Predicting functional profiles with Tax4Fun2

In the initial step of Tax4Fun2, user-supplied 16S rRNA gene sequences (operational taxonomic units or exact sequence variants [50], also known as zero-radius OTUs, but here simply referred to as OTUs) are searched against the 16S rRNA reference sequences by BLAST using the *runRefBlast* function (Fig. 1). Other tools, such as paprica [26], use algorithms to place query sequences into phylogenetic trees. These algorithms usually provide a very accurate phylogenetic placement. However, placing sequences is very hardware intensive and most algorithms are restricted to Unix or Unix-like operating systems. Due to these limitations, we decided to use a BLAST-based approach, because we only need to identify the closest match in the database. If user data is supplied, the next-neighbour search is repeated using the user-generated reference data. Following the assumption that users provide habitat or site-specific data, user-generated data is preferred to the default reference data. Specifically, if the next neighbour search for one OTU against both the default reference database and the user-generated database resulted in significant hits, the user data is incorporated in the functional prediction.

Functional predictions are subsequently calculated using the *makeFunctionalPrediction* function (Fig. 1). During this step, the OTU table supplied by the user is summarized based on the results of the next neighbour search. A specific association matrix in the summarized table is generated containing only the functional reference profiles of the next neighbours. For each sample, the abundance information from the OTU table and the functional information stored in the specific association matrix are converted into a sample-specific functional profile. Predicted profiles are later summarized based on KEGG pathways. Only OTUs passing a defined similarity threshold (default = 97%) are considered in the functional prediction. The fraction of OTUs having no close hit in the reference data and hence are unused in the subsequent prediction (fraction of taxonomic units unused = FTU) as well as the amount of sequences assigned to these unused taxonomic units (fraction of sequences unused = FSU) is recorded. FTU and FSU values may serve as an additional quality indicator for the predicted metagenomes as high FTU and/or FSU values indicate that predictions were made only for a small fraction of the total microbial community.

#### Calculation and validation of the functional redundancy index (FRI)

**Fig. 2:**
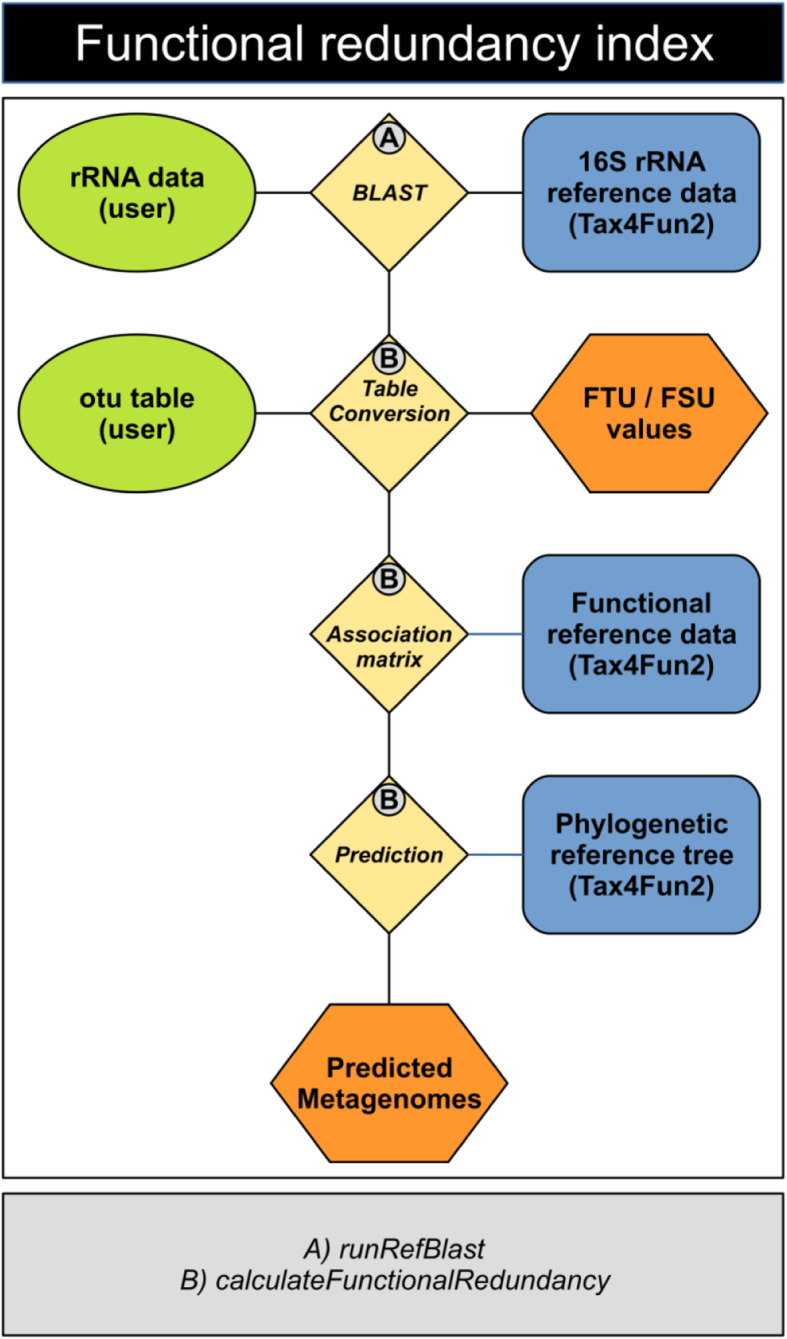
The FRI workflow. 16S rRNA gene sequences are initially aligned against the reference sequences provided with Tax4Fun2 to identify the nearest neighbour. The OTU abundances for each sample are converted to presence-absence data and subsequently summarized based on the results from the nearest neighbour search. An association matrix (AM) containing the normalized functional profiles of those references identified in the 16S rRNA search is generated. The normalized functional profile in the AM is multiplied by the summarized OTU abundance. We assume that a function is present if the abundance of a function is greater than 50%. For instance, if 5 OTUs were affiliated to one 16S rRNA reference seqeunce, we would assume that every function with a minimum abundance of 10% in the normalized reference profile would be present in the AM. The FRI is finally calculated based on those community members possessing the function and their phylogenetic distribution. The amount of sequences/OTUs unused in the prediction (FSU/FTU) is provided in a log file 16S rRNA gene sequences are initially aligned against the reference sequences provided with Tax4Fun2 to identify the nearest neighbour. The presence of each OTU are summarized based on the results from the nearest neighbour search. An association matrix (AM) containing the functional profiles of those references identified in the 16S rRNA search is generated. The summarized abundances and the functional profiles stored in the AM are multiplied. Functions being present in all genomes are assumed to be present in the entire cluster. The functional redundancy is subsequently predicted from the average phylogenetic distance of all community members potentially possessing the function. This distance is subsequently normalized either by the average phylogenetic distance of the total reference tree (absolute) or by the distance found in the surveyed community (relative). FTU and FSU values are provided as a log file. The functions performing each step are marked with numbers

To date, there is no tool available to predict functional redundancies based on 16S rRNA data. Here, we introduce the functional redundancy index (FRI), which describes the (multi-) functional redundancy of a prokaryotic community, i.e., the redundancy of multiple functions in the investigated community. The FRI incorporates the phylogenetic distribution (distance) of community members harbouring a specific function and their proportion in the community. In Tax4Fun2, the functional redundancy index is calculated using the function *calculateFunctionalRedundancy* (Fig. 2).

Following next neighbour search, the OTU table is summarized based on the results of the next neighbour search and converted to binary data (presence/absence). The normalized functional profile associated with each reference sequence is also converted to a binary data (presence/absence) using a cut-off of 100% meaning that a function is considered to be present if it was observed in all genomes assigned to the reference profile. If the next neighbour search identifies the same neighbour for several OTUs, then the normalized functional profile associated with the 16S rRNA reference sequence is multiplied by the number of OTUs before it is being converted to binary data. This is based on the assumption that the probability a specific function present in a community is higher the more distinct the phylotypes associated to a single reference are present. The redundancy of any function present in the community is subsequently calculated by multiplying the average phylogenetic distance of all community members possessing the function with their proportion in the community. The product is normalized by the average phylogenetic distance of the total community. To account for differences in average phylogenetic distances between different communities, we provide the possibility to calculate the absolute and the relative FRI (aFRI and rFRI). To calculate the aFRI, the average phylogenetic distance of all species in the full 16S rRNA reference tree is used for normalization, whereas the rFRI is normalized by the average phylogenetic distance of those species in the 16S rRNA reference tree identified as being present in the surveyed samples during next neighbour search. The rFRI can be used to compare samples within one survey, whereas the aFRI allows the comparison of functional redundancy indices across different ecosystems. The latter is important as multifunctional redundancy comparisons between or among different environments generate a more robust depiction of (regional) variation in the resilience/vulnerability of microbial communities [21].

To test the FRI accuracy, we simulated 1000 prokaryotic communities each consisting of 100 genomes randomly selected from the 12,377 genomes used to generate the reference data. To assess the phylogenetic distance between the genomes, we extracted 63 marker protein sequences based on hmm profiles derived from PFAM version 31 [51] and TIGRFAM version 15 [52]. The 63 marker proteins were selected because their corresponding genes were present in 90% of all 12,377 genomes and, if present, were single-copy genes in 99% of them. These criteria were applied to archaea and bacteria independently. The extracted protein sequences of each hmm profile were aligned using mafft version 7.3.11 [53]. Afterwards, aligned protein sequences for each genome were concatenated. The phylogenomic tree was calculated using FastTree version 2.1.10 [54]. Functional profiles for each genome were converted to presence-absence data and the FRI was calculated for each function using the genome tree and the presence-absence data. The 16S rRNA gene sequences present in each genome subset were separately clustered in operational taxonomic units (OTUs) at 97% similarity with UCLUST implemented in USEARCH [41]. An OTU table was generated based on the clustering. Each OTU was represented by its longest sequence.

## Results and discussion

### Tax4Fun2 evaluation

We first applied Tax4Fun2 in comparison to Tax4Fun [23] and PICRUSt [22] using the same paired samples (16S rRNA gene and metagenome data), which were used to validate both tools, i.e. samples derived from the human microbiome, mammalian guts, soil and from a hypersaline microbial mat (Table 1), in addition to ten marine seawater samples taken in the North Sea [7] and 90 kelp-associated samples collected within the Marine Microbes Framework Data Initiative (http://www.bioplatforms.com/marine-microbes).

**Table 1:** Accession numbers of samples/studies used to validate Tax4Fun2

Origin	Sample number	Accession numbers
Human Microbiome	41	SRS011271, SRS011452, SRS011529, SRS011584, SRS011586, SRS013234, SRS013252, SRS013258, SRS013506, SRS013687, SRS013711, SRS013723, SRS014235, SRS014287, SRS014343, SRS014613, SRS014629, SRS014923, SRS015133, SRS015190, SRS015425, SRS015450, SRS015574, SRS015578, SRS015762, SRS015782, SRS015854, SRS015960, SRS016002, SRS016018, SRS016095, SRS016111, SRS016203, SRS016225, SRS016331, SRS016335, SRS016349, SRS016434, SRS016533, SRS016553, SRS016559
Mammalian Gut	56	4,461,284–301, 4,461,341–55, 4,461,357–58, 4,461,360–80, 4,461,383 (MG-RAST)
Microbial Mat	10	4,440,963–71 (MG-RAST)
Soil	14	4,477,803–5, 4,477,807, 4,477,872–7, 4,477,899, 4,477,902–4 (MG-RAST)
Water	10	SRA060677
Kelp	90	57,884–936, 57,938–56, 87,958–74, 58,019–20 (https://data.bioplatforms.com/organization/about/australian-microbiome).)

Functional profiles were predicted using the default workflows. For PICRUSt, processed sequences were clustered using QIIME version 1.8 [55] by closed-reference picking against the Greengenes database (version 13_5; [56]) and normalized prior to functional prediction. For Tax4Fun, OTU sequences were taxonomically classified by BLAST search [57] against the SILVA database (SILVA_123_SSURef_Nr99) [46].

**Fig. 3:**
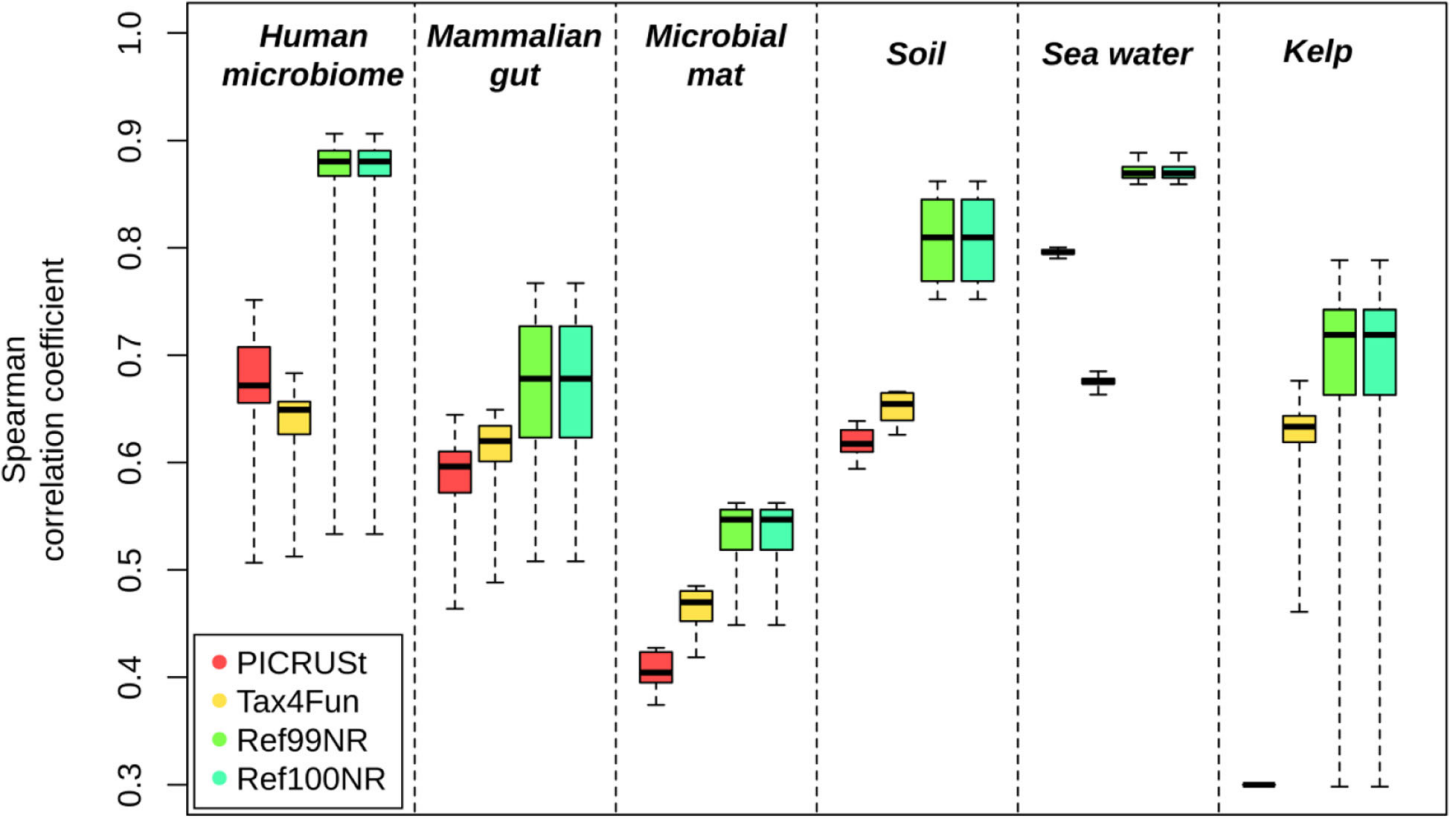
Correlations between functional profiles obtained from metagenomic datasets and those predicted from 16 s rRNA data. Predictions were made with PICRUSt, Tax4Fun, and Tax4Fun2. Predictions with Tax4Fun2 were made using both supplied default reference datasets (Ref99NR and Ref100NR). Note that PICRUSt did not generate any prediction for the kelp data

We evaluated the predictive power of each tool by comparing the functional profiles predicted from the 16S rRNA data to functional profiles generated directly from the metagenomes using Spearman rank correlations. Comparing the profiles predicted with PICRUSt, Tax4Fun and Tax4Fun2 with metagenome-derived profiles clearly showed that Tax4Fun2 outperforms PICRUSt and Tax4Fun across all six tested datasets (Fig. 3). In addition, Tax4Fun2 was more than 20 times faster than Tax4Fun due to the smaller reference database.

It should be noted that a direct comparison of functional profiles predicted with all three tools is difficult due to several changes in the KEGG Orthology since PICRUSt and Tax4Fun were developed (deprecated and new functional orthologs). Hence, predicted functional profiles as well as those obtained by metagenomic shotgun sequencing were converted to relative abundances prior to comparison. Only functions present in the metagenomic profile and in the predicted profile were considered in each comparison. On average, more than 95% of all functions in the human microbiome as well as the marine, soil and kelp-derived metagenomes were affiliated to these shared KOs. In the microbial mat and the mammalian gut samples, more than 74 and 78% of all predicted functions were affiliated to these KOs, respectively.

### Using user-defined data increased the accuracy and reduced the FTU

Following the assumption that any additional genomic information specific for the investigated habitat further enhances the predictive power of Tax4Fun2, we used 68 metagenome-assembled genomes (MAGs) generated from the 90 kelp-associated metagenomes to build a kelp-specific reference dataset. These genomes were selected because at least one 16S rRNA gene sequence was identified in each genome.

**Fig. 4:**
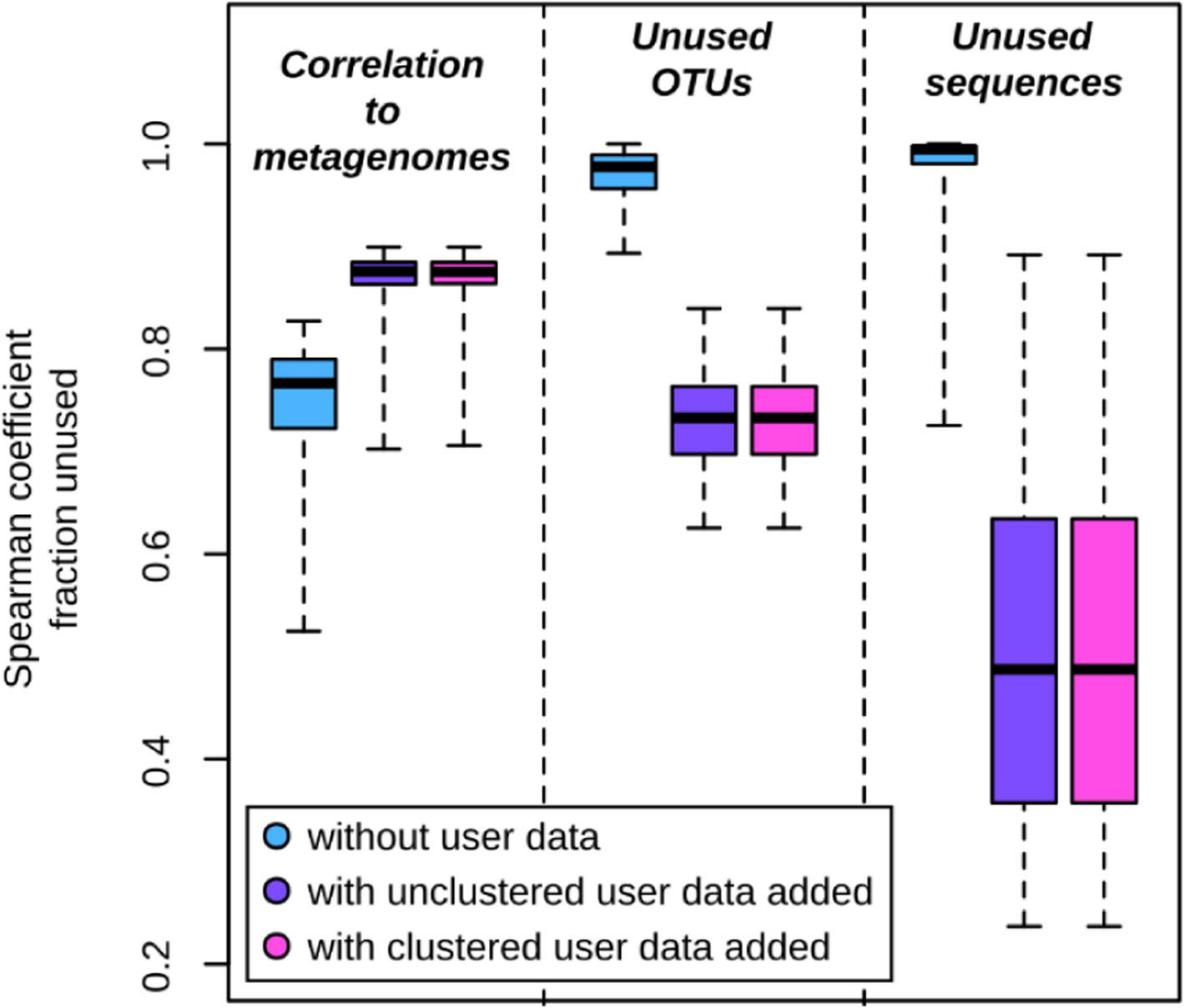
Correlations between functional profiles retrieved from 90 kelp metagenomes and those predicted with Tax4Fun2 without and with user data added and the fraction of zOTUs and sequences unused in the prediction

Using the default data, the median Spearman correlation coefficient was 0.72. Incorporating the kelp-specific data substantially increased the power of the functional prediction (median Spearman correlation coefficient = 0.86) and reduced the fraction of the sequences not used in the prediction (Fig. 4), showing that a lack of suitable reference genomes did initially limit Tax4Fun2’s performance. Moreover, using the kelp-specific dataset enabled us to predict functional profiles for samples, which failed when using only the default reference data, because next neighbour search resulted in no close matches. These results demonstrate the benefits of incorporating user-defined, habitat-specific reference databases, which distinguishes Tax4Fun2 from all other published tools.

### Functional redundancy index

The simultaneousness assessment of multiple functions is usually very time-consuming [20]. Previous studies have focused on a limited number of community functions (e.g., [58–60]). However, the degree of functional redundancy in any given system depends on the functions considered [14]. In addition, it is difficult to draw conclusions about functional redundancy based on a single environmental situation, as species that are functionally redundant in one environment might be pivotal in another [61]. Hence, the extent of functional redundancy change as the ecological contribution of a species might change between different environments. Nonetheless, a contemporary concern for the conservation of biodiversity and the development of management strategies is that decision makers require quantitative measures as part of science-based negotiations and communications. In order to provide those measures when assessing natural or human-induced impacts on an ecosystem, we introduced the FRI with respect to multiple functions in Tax4Fun2. A high FRI indicates that a specific function is almost ubiquitous in all community members, whereas a low FRI suggests that the function is present in a few closely related species. A FRI of 0 indicates that a function has been detected in only one community member or is not present at all. Consequently, the lower the FRI the higher the probability that a function gets lost after community shifts or perturbations.

**Fig. 5:**
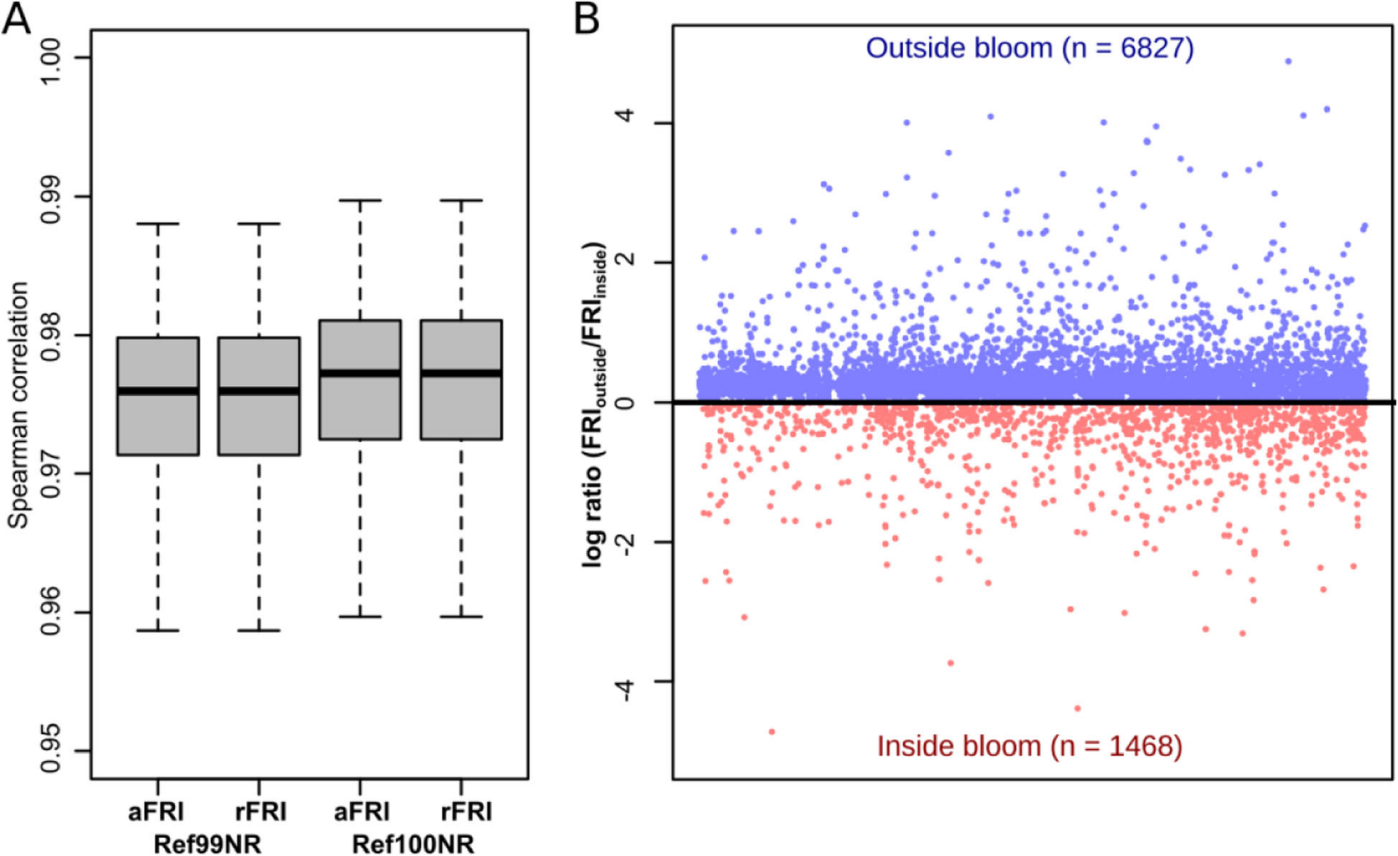
Correlation between predicted and genome-based functional redundancy indices (**a**) and functional redundancy indices inside and outside a phytoplankton bloom (**b**). A log ratio greater than 0 indicates that a function is more redundant outside the bloom. All predictions were made using a 97% similarity cut off

To test the accuracy, we simulated 1000 microbial communities and calculated FRI values based on 16S rRNA gene data using Tax4Fun2. The FRIs calculated for each function were subsequently compared to the FRIs calculated directly from the genomes of each simulation by Spearman rank correlation. The comparison revealed that Tax4Fun2 provides a good estimate of the functional redundancy present in the microbial community (Spearman rank correlation > 90%) (Fig. 5a).

We further calculated FRIs using the marine seawater samples. Six of these samples were taken inside a phytoplankton bloom and three samples were taken outside the bloom [7]. Nearly 7000 functions displayed a higher functional redundancy index outside the bloom, whereas only 1468 functions had higher redundancies inside the bloom (Fig. 5b). This indicates that the functional redundancy greatly shifts during the phytoplankton bloom. Phytoplankton blooms are usually characterized by a substrate-controlled succession, i.e. distinct bacterial clades dominate the bacterioplankton community at different stages during and shortly after the bloom [62]. Consequently, community members involved in the turnover of certain substrates at a specific stage are predominant. For instance, the SAR92 clade, the *Roseobacter* RCA cluster and the genus *Polaribacter* were more abundant in bloom samples [3]. Because we did not observe significant differences in the phylogenetic diversity of bacterioplankton communities derived from bloom and reference samples, functions predominantly associated with dominant community members are more redundant in the bloom whereas all other functions display higher redundancies in the reference samples.

## Conclusion

With Tax4Fun2, we provide an easy-to-use, platform-independent R package, which enables researchers to predict and investigate functional profiles of prokaryotic communities based on 16S rRNA gene data. We demonstrate the high predictive power of Tax4Fun2, providing superior results to any other established tool. The key strength of Tax4Fun2 is the incorporation of user-defined and habitat-specific data, which further enhances the accuracy of the predictions. Another unique feature of Tax4Fun2 is that it enables researchers to calculate functionial redundancies, which is a relevant parameter for ecosystem monitoring and the development of management strategies to safeguard optimal ecosystem functionality. Nonetheless, functional profiles and functional redundancies are predictions only and should be treated with caution.

### Availability and requirements

Project name: Tax4Fun2.

Project homepage: https://github.com/bwemheu/Tax4Fun2

Operating system(s): Platform-independent.

Programming language: R.

Other requirements: BLAST+ 2.7.1 or later, R packages ape and seqinr.

License: GNU General Public License v3.0.

Any restrictions to use by non-academics: no.

## Data Availability

The dataset(s) supporting the conclusions of this article is available at https://zenodo.org/records/10035668.

## Abbreviations

BLAST: Basic Local Alignment Search Tool
NCBI: National Center for Biotechnology Information
FRI: Functional redundancy index
